# Predictors of Delirium in Octogenarian Patients Hospitalized for a Hip Fracture

**DOI:** 10.3390/ijerph17207467

**Published:** 2020-10-14

**Authors:** María Plaza-Carmona, Carmen Requena-Hernández, Sonia Jiménez-Mola

**Affiliations:** 1 Geriatrics Unit, León University Hospital, 24008 León, Spain; soniajimenezmola@hotmail.com; 2 Department of Psychology, Sociology and Philosophy, University of León, 24071 Leon, Spain; c.requena@unileon.es

**Keywords:** delirium, hip fracture, elderly, hospitalization

## Abstract

Introduction: Since delirium is a major complication that can arise after a patient with a hip fracture has been hospitalized, it is considered to be one of the most common geriatric conditions. Therefore, its prevention and early detection are essential for reducing both the length of the patient’s stay in the hospital and complications during the hospitalization process. Objective: To identify and analyze the predictors for developing delirium in octogenarians who were admitted to hospital for a hip fracture. Methodology: A prospective study conducted with a sample of 287 patients aged 80 years and older (mean age 87.2 ± 3.2 years; 215 women, 72 men), recruited from the Trauma Unit of the University Hospital of León (Spain). Further, 71.1% of the patients lived in a family member’s home, while the other 28.9% lived in a nursing home. After observing each patient’s interactions with their doctor in a clinical setting, the data for this study were obtained by reviewing the selected patients’ charts. The variables analyzed were sociodemographic information (age, sex, and place of residence), medical information (type of hip break and surgical intervention), cognitive impairment (MMSE score), functional level (Barthel Index score), and clinical information (pharmacological, comorbidities, complications, and the diagnosis and assessment of the severity of delirium in a patient). The univariate and multivariate logistic regression analysis showed a significant relationship between acute confusional state and the following variables: anemia, American Society of Anesthesiologists (ASA) III and IV patients, state of cognitive frailty and functional level, a urinary tract infection, changes in the visual field, renal arterial occlusion, and the type and dosage of drugs administered (this variable was identified in the multivariate model). The inverse relationship between anemia and acute confusional state is surprising. Conclusion: This research shows that clinical observation of acute confusional state is necessary but not sufficient for addressing this condition early and adequately in older adults who have been hospitalized for a hip fracture.

## 1. Introduction

Delirium is an acute and fluctuating syndrome, characterized by a change in attention and level of consciousness and by cognitive dysfunction, which develops in a short period of time and cannot be explained solely by the preexistence or development of dementia [1]. There are three types of clinical presentation of delirium: hyperactive (patients present hyperactivity, agitation, aggressiveness, confusion, hallucinations, and delirious ideation), hypoactive (patients present hypoactivity, somnolence, psychomotor slowing, slow language, and apathy), and mixed (patients present characteristics of both) [2,3]. It is the second most prevalent psychiatric condition in older people and the fourth most common complication to occur in patients hospitalized for a hip fracture [2].

Prevention and rapid identification of the variables associated with delirium are essential for reducing the length of a patient’s hospital stay, decreasing the demand for social and medical resources during their hospital stay, and improving their health as quickly as possible [4]. This can also lead to a decrease in how powerless nursing assistants feel when they take care of these patients’ personal hygiene (e.g., helping them shower and changing their bedsheets). It has been found that in the general population, the prevalence of delirium during a patient’s hospital stay is 18–34% and the incidence is 29–64% [5]. If you analyze the existing data on different geriatric services, you can see that the prevalence of delirium is 20–29% in geriatrics and 12–51% in orthopedic surgery. Likewise, it has been estimated that the presence of delirium after a hip fracture operation presents in between 4% and 53% of cases; consequently, knowing the variables associated with the syndrome will allow for an accurate diagnosis [6].

Delirium has a multifactorial origin, so establishing its physiopathology solely from its etiology and clinical presentation is complicated. Some research points to the hypothesis of oxidative stress as one of the most plausible medical theories establishing delirium’s physiopathology [7]. It explains how a decrease in oxidative metabolism in the brain results in a brain dysfunction. Likewise, it is important to point out the neuronal aging hypothesis, which establishes that the elderly have a higher risk of developing delirium as a consequence of the increased number of changes that occur in their stress-regulating neurotransmitters and in their intracellular signal transduction systems [7,8].

In turn, the combination of risk factors, predisposing factors, and precipitants presented by the elderly during their hospital stay make them more vulnerable to developing this condition [5,9,10,11]. In particular, the number of predisposing factors a patient has is an essential component of predicting the development of delirium, especially in older and frail people [9].

Among the risk factors that have been described as being present in acute care units and intensive care units are polypharmacy; surgery that is not carried out carefully; and the patient’s Barthel Index, urea/creatinine ratio, level of cognitive impairment, and mobility [4,10,11]. It is also important to note how the presence of comorbidities is associated with increased risk at all ages [5]. Additionally, the latest studies show how the frailty of older patients can be a predisposing factor for developing delirium [12]. Moreover, it is necessary to mention that monitoring delirium with validated screening tools (e.g., CAM-ICU, NuDESC, ICDSC, and others) enables early identification of delirious patients, especially those who develop hypoactive delirium [13]. This is why the European Society of Anesthesiology establishes that delirium should be monitored until the fifth postoperative day [14].

Therefore, the objective of this study is to analyze predictors of delirium during the hospital stay of octogenarian patients who were hospitalized for a hip fracture, with the aim of reducing health care costs and increasing the safety of health professionals at work.

## 2. Materials and Methods

A prospective cohort study. The study sample was established from September 2019 to February 2020. The inclusion criteria were patients aged 80 years and older who were to have surgery for their hip fractures during the months established for the study. The exclusion criteria were patients with a pathological hip fracture caused by anything other than osteoporosis and patients with a hip fracture caused by a traumatic fracture. The data were extracted from the clinical histories of the patients during their hospital stay. The medical records collected from each patient revealed the most relevant data for this study since they included clinical observations made while the doctor was interacting with the patient. As a statistical technique, a linear regression analysis (univariate and multivariate) was applied to the data to study the relationship between acute confusional state and the following variables: sociodemographic information (age, sex, and place of residence), surgery information surgical (type of fracture, type of prosthesis, anesthesia used), medications (pre-admission and drug administration), comorbidities, complications (clinical complications after surgery, anemia, and high blood pressure), and the patient’s level of cognitive and functional impairment (following the MMSE criteria and Barthel Index criteria, respectively). The diagnosis and assessment of the severity of delirium in patients was made after carefully observing and watching for the most relevant characteristics of the syndrome, such as its abrupt onset, its symptoms’ tendency to fluctuate, changes in global cognitive functioning, and, in particular, abnormal performance when it comes to attention, orientation, and organizing thoughts [15].

The study followed the guidelines for observational studies in epidemiology (STROBE), the Deontological Standards recognized by the 1975 Declaration of Helsinki (revised at the 52nd General Assembly in Edinburgh, Scotland, October 2000), and the Standards of Good Clinical Practice. It complied with Spanish legislation and legal regulations governing clinical research in humans (Royal Decree 223/2004 on the regulation of clinical trials). The project was approved by the Clinical Research Ethics Committee of the Hospital of León and received the approval of the Ethics Committee (the code of ethics for research is 16109, dated 29 November 2016).

The statistical analyses were analyzed using the SPSS statistical package for Windows (SPSS v 22.0. Inc., Chicago, IL, USA), which established the significance level at *p* < 0.05.

Descriptive data were presented as mean values, quantitative standard deviation (SD) variables, and qualitative variables of percentages and frequencies. The significance of the association was estimated by means of the Chi-square test of independence, accompanied by the size of the effect using R2 calculated from Cramer’s V. The predictive capacity of the factor was analyzed using multivariable logistic regression analysis. Odd ratios were calculated to compare the influence of the explanatory variables on the presence/absence of the factor (acute confusional state). Finally, the predictive capacity of the factor was calculated again using R2 estimated from the Nagelkerke index.

## 3. Results

The study involved a total of 287 orthogeriatric patients recruited from the Trauma Unit of the University Hospital of León (Spain). The mean age was 87.2 ± 3.2 years. There were 215 women (74.9%: 95% CI: 69.5%–79.8%) and 72 men (25.1%; 95% CI: 20.2%–30.5%). Further, 71.1% of all the study participants lived with a family member (39.7% of the patients lived with someone and the other 31.4% lived alone) and 28.9% of the participants lived in a nursing home. The results found show that cognitive impairment (MMSE) is significantly related to the development of delirium (severe cognitive impairment *p* = 0.004; moderate cognitive impairment *p* = 0.002). The Barthel scores (ranging from total to mild dependency) from the sample show that the Barthel Index is not significantly related to delirium. However, the data we have seem to indicate a greater presence of delirium cases among patients with total dependency (47.8%) compared with the remaining classifications (between 30.4% and 37.1%). Something similar occurs with ambulation ability since the variable, as initially defined, does not reach significance. However, in view of the data, there are more cases of delirium (50.0%) in patients who do not walk than among patients who do (Table 1).

Comorbidities (heart disease, hypertension, depression, atrial fibrillation, arthrosis, and diabetes) are not significantly related to delirium. A statistically significant relationship does appear (*p* < 0.05) with visual impairment, such that patients suffering from it tend to have a higher chance of suffering from acute coronary syndrome (ACS) (44.8% vs. 29.7%; OR = 1.92). Additionally, dementia appears to be a very significant variable related to delirium (*p* < 0.01); therefore, patients with dementia are more likely to present with ACS (46.7% vs 27.8%; OR = 2.27). Consequently, we can affirm that these variables (visual impairment and dementia) are effective predictors of delirium (Table 2).

No significant relationship was found between delirium and the type of fracture, the type of anesthesia used, or the type of surgery employed. However, statistical significance (*p* < 0.05) was found in American Society of Anesthesiologists (ASA) values, particularly in grades III and IV (36.3%, OR = 1.95) as compared to grades I and II (22.7%). Consequently, ASA category classifications of III and IV are effective in predicting delirium (even though their effects range from mild to moderate (2.4%) (Table 2).

The type and amount of medication (antihypertensives, benzodiazepines, antidepressants, proton pump inhibitors, antiaggregants, anticoagulants, oral antidiabetics, and analgesics) are not related to delirium. In contrast, anemia and a urinary tract infection do show significant differences, *p* < 0.05 and *p* < 0.001, respectively. In particular, the anemia data show an inverse significant relationship with the syndrome. That is, the lower the anemia, the greater the risk that older people will develop this syndrome (30.5% developed delirium, while 47.4% did not; OR = 0.48), with a medium-low predictive value (2%). On the other hand, the urinary tract infection data show that 63.4% of patients with a UTI developed delirium, while 27.6% did not (OR = 4.54), with a moderate-high predictive value (9%). Likewise, a significant association between renal arterial occlusion (RAO) and delirium has been found (*p* < 0.01). In particular, the greater the risk for delirium, the higher the chance of developing RAO (58.1% vs. 29.7%; OR = 3.28); the predictive value for the presence of delirium is moderate (4.5%). The rest of the variables, such as transfusion, constipation, respiratory function, renal function, malnutrition, chronic heart failure (CHF), and ischemic cardiopathy, do not reach statistical significance (*p* > 0.10) (Table 3).

As can be seen in both Table 3 and Table 4, the results of the univariate and multivariate linear regression models show that the same factors are associated with the risk of developing acute confusional state. In addition, Table 4 identifies the number of drug administration as another factor associated with this syndrome.

When analyzing the drugs administered to help with delirium symptoms, no relationship was observed between the different types of medication and delirium (Table 5). In addition, none of the medications were statistically related to the presence of delirium (*p* > 0.01), nor could they be considered as possible effective predictors of the syndrome (effects *p* < 0.01). Only the anti-hypertension drugs were relatively close to the limit of significance that we established (*p* = 0.110) due to the fact that those who take them are less prone to developing it (29.8% vs 39.7%); so, these data could be pointing to the possibility of considering this variable as a protective factor for delirium.

## 4. Discussion

The symptoms of delirium are numerous and variable and fluctuate; recognizing them is important since their early diagnosis can reduce how much a patient suffers from the syndrome and lower costs for the health care system. There are concrete data that show the economic importance of adequately managing this disorder in elderly patients with a hip fracture. The mean length of a patient’s hospital stay changing from 10.6 to 6.9 days and the mean waiting time for fracture intervention changing from 3.5 to 1.1 days represents an improvement in care, an optimization of resources, and an estimated cost reduction per treated fracture of 30% [16].

This research studied the predictive clinical variables of delirium in 287 patients (69.5% women and 30.5% men), of whom 71.1% lived in a family member´s home and 28.9% lived in a nursing home. The fact that the sample in our study had a higher ratio of women to men is not a characteristic of just our study; in general, this proportion is common in both clinical and social research. One possible explanation is that for cultural reasons, women are usually more open to participating in research studies than men and in clinical contexts, it can be explained by the fact that women have a higher life expectancy than men [17].

The type of fracture that we observed to have occurred most frequently among our study participants was a pertrochanteric fracture (155 patients) (54%; CI: 48.1–59.9%); the rest of the patients had a subcapital fracture (132 patients) (46.0%; CI: 40.1–51.9%). This coincides with recent studies that show that 48% of patients presented the first type of fracture just mentioned [12]. The type of anesthesia given to patients was primarily spinal (87.5%), as has been recommended for geriatric patients [18]. The results of the univariate and multivariate models applied in this study show that the presence of anemia, a grade of III or IV (based on the criteria of the American Society of Anesthesiologists (ASA) Physical Status Classification System), a patient’s cognitive and functional level, a urinary tract infection, visual disturbances, a renal arterial occlusion, and the drugs administered to a patient during their hospital stay, in this order, can be considered as predictive variables for delirium in octogenarian patients who are hospitalized for a hip fracture. The presence of anemia appears to be a protective factor for delirium. This is of great interest because anemia is a common complication that arises while a patient is recovering from a hip fracture, but it has not been described as a protective factor [4,5,6,7,8,9,10,11,19].

At present, one of the most powerful models used to integrate the different risk factors for delirium is based on the concept of “brain reserve” [20]. This concept refers to the central nervous system’s great ability to respond in a functionally flexible way to aggressions. In our study, most of the patients had a meager brain reserve because of cognitive impairment, which would explain their predisposition to delirium.

The data from our study relate visual deficit to delirium and drug administration. Other investigations do this as well, such as the one carried out by Inouye et al. [21]. In their study, they showed that acting on variables that are expected to increase the risk of developing delirium could reduce the number and duration of episodes of this syndrome in hospitalized patients. Among the variables that were acted upon were symptoms of sensory deprivation, drug administration, and the patients’ level of functionality. The results show that delirium appeared in 9.9% of the patients for whom these variables were controlled compared to the 15% of the patients for whom these variables were not controlled. Despite the fact that our results do not show an association between a patient’s functional level and delirium, it is observed that subjects with less functional autonomy have a greater risk of developing delirium (Table 2).

Regarding the comorbidities studied (heart disease, hypertension, depression, atrial fibrillation, arthrosis, poorly controlled diabetes, and thyroid disorders (hypo or hyperthyroidism)), none of them show a significant relationship with delirium. These data contradict the results of other investigations, such as the one carried out by Kotfis et al., 2018 [22], with patients admitted to internal medicine (where ischemic heart disease and atrial fibrillation are the most common symptoms). On the other hand, the study carried out by Oh et al. [23] shows that endocrine diseases, such as poorly controlled diabetes and thyroid disorders (hypo or hyperthyroidism), precipitate towards acute confusional state. The lack of consistency of our results with the indicated investigations could be related to the age of the subjects in our study. Note that only 47.4% of our subjects showed cognitive impairment, 8% had a low functional level, and 98.2% had a comorbidity. These data show that the health of our research subjects was better than that of the subjects of the referenced studies. In addition, 32.2% of our subjects indicated that their place of residence was a family member´s home, while 37.3% indicated that they lived in a nursing home. Some research indicates that 60% of people who live in nursing homes develop acute confusional state [24,25] compared to 1–2% of subjects who live at home [26].

Depression in hospitalized patients increases with age and in older people, it is the most prevalent disorder (10–56% of hospitalized patients), followed by acute confusional state (10–30%) [27]. In our study, 32.4% of the patients suffered from depression, but the data were obtained through clinical observation and a specific instrument was not applied that allowed a differential diagnosis between depression and delirium. Therefore, we could conjecture that the diagnoses of depression could actually be cases of hypoactive delirium; people with this type of delirium show a decrease in psychomotor activity, which can be mistakenly diagnosed as depression [28].

The study has limitations related to its methodology. On the one hand, the delirium data were obtained through clinical observation, so the information collected from the patients’ medical records depended on the expertise of the professional who collected the data and not on objectified data. On the other hand, the research design was cross-sectional, so there are no follow-up data on the patients. However, despite these limitations, the data reported are consistent with other studies conducted with older people who were hospitalized for a hip fracture (e.g., physiological, sensory, and functional variables appear to be associated with delirium).

## 5. Conclusions

The diagnosis of acute confusional state is eminently clinical, but the observation of the doctor is not enough. Therefore, it is necessary to implement a protocol designed to collect information on the clinical manifestations of delirium that recognizes and prevents the predisposing and triggering factors of this syndrome. This would make it possible to start adequate treatment before severe cognitive impairment develops.

## Figures and Tables

**Table 1:** Test of independence and univariate logistic regression. Effect of patient baseline factors on the presence of delirium in orthogeriatric patients with a hip fracture.

Factor	Description of Patients with Delirium	Contrast Test		R^2^	Univariate Logistic Regression				
		Valor	*p*		OR	95% CI	Wald	*p*	R^2^
**BARTHEL**									
Total dependency	47.8% (11)	3.19 ^NS^	0.363	0.011	2.10	0.88/5.00	2.78 ^†^	0.096	0.015
Severe dependency	37.1% (13)				1.35	0.64/2.85	0.62 ^NS^	0.430	--
Moderate dependency	31.8% (7)				1.07	0.42/2.74	0.02 ^NS^	0.893	--
Slight dependency	30.4% (63)				1	--	--	--	--
Independent	31.4% (83)				1	--	--	--	--
**DEAMBULATION**									
Does not walk	50.0% (7)	2.92 ^NS^	0.403	0.010	2.10	0.70/6.27	1.78 ^NS^	0.183	0.014
Needs a lot of help	39.1% (9)			Ht	1.35	0.55/3.30	0.44 ^NS^	0.509	--
Walker/two canes	28.4% (19)				1.20	0.65/2.22	0.34 ^NS^	0.558	--
Independent/one cane	32.2% (59)				1	--	--	--	--
Does not walk/A lot of help	43.2% (16)	2.12 ^NS^	0.145	0.007	1.68	0.83/3.39	2.09 ^NS^	0.148	0.010
Walker/Independent	31.2% (78)				1	--	--	--	--
Walker/Independent	50.0% (7)	1.99 ^NS^	0.159	0.007	2.14	0.73/6.28	1.91 ^NS^	0.167	0.009
Does walk (with help)	31.9% (87)				1	--	--	--	--
**PLACE OF RESIDENCE**									
Nursing home	37.3% (31)	1.25 ^NS^	0.535	0.004	1.40	0.77/2.55	1.23 ^NS^	0.268	0.006
Lives with family	32.2% (29)				1.12	0.62/2.03	0.14 ^NS^	0.713	--
Lives alone	29.8% (34)				1	--	--	--	--
**COGNITIVE DETERIORATION**									
Severe	7.1% (1)	26.44 **	0.000	0.092	0.23	0.03/1.81	1.96 ^NS^	0.162	0.123
Moderate	57.8% (37)				4.08	2.20/7.56	19.89 **	0.000	
Mild	31.0% (18)				1.34	0.69/2.61	0.73 ^NS^	0.392	
No deterioration	25.2% (38)				1	--	--	--	--
Slight/no deterioration	26.8% (56)				1	--	--	--	--

NS = NOT significant at 10% (*p* > 0.100); ^†^ = Significant cases (*p* < 0.100); ** = Highly significant at 1% (*p* < 0.01).

**Table 2:** Test of independence and univariate logistic regression. Effect of the factors of the most frequent comorbidities (>20%) and factors related to IQ on the presence of delirium in orthogeriatric patients with a hip fracture.

Factor	Description of Patients with Delirium	Contrast Test		R^2^	Univariate Logistic Regression				
		Valor	*p*		OR	95% CI	Wald	*p*	R^2^
**CARDIOPATHY**									
Yes	32.1% (67)	0.17 ^NS^	0.681	0.001	0.89	0.51/1.54	0.17 ^NS^	0.681	0.001
No	34.6% (27)				1	--	--	--	--
**HIGH BLOOD PRESSURE (HBP)**									
Yes	30.5% (61)	1.52 ^NS^	0.218	0.005	0.72	0.42/1.22	1.51 ^NS^	0.218	0.007
No	37.9% (33)				1	--	--	--	--
**DEPRESSION**									
Yes	35.5% (33)	0.47 ^NS^	0.495	0.002	1.20	0.71/2.02	0.47 ^NS^	0.495	0.002
No	31.4% (61)				1	--	--	--	--
**DEMENTIA**									
Yes	46.7% (35)	8.92**	0.003	0.031	2.27	1.32/3.91	8.71**	0.003	0.041
No	27.8% (59)				1	--	--	--	--
**ATRIAL FIBRILLATION**									
Yes	35.3% (24)	0.26 ^NS^	0.609	0.001	1.16	0.66/2.06	0.26 ^NS^	0.609	0.001
No	32.0% (70)				1	--	--	--	--
**ARTHROSIS**									
Yes	29.9% (20)	0.33 ^NS^	0.563	0.001	0.84	0.46/1.52	0.33 ^NS^	0.563	0.002
No	33.6% (74)				1	--	--	--	--
**DIABETES**									
Yes	31.8% (21)	0.03 ^NS^	0.854	0.000	0.95	0.52/1.70	0.03 ^NS^	0.854	0.000
No	33.0% (73)				1	--	--	--	--
**CHANGES IN VISION**									
Yes	44.8% (26)	4.81 *	0.028	0.017	1.92	1.07/3.47	4.72 *	0.030	0.022
No	29.7% (68)				1	--	--	--	--
**TYPE OF TREATMENT**									
Bipolar partial prostheses	37.0% (27)	3.28 ^NS^	0.512	0.012	1.37	0.75/2.49	1.07 ^NS^	0.302	0.017
Total prosthesis	50.0% (8)				2.33	0.82/6.63	2.53 ^NS^	0.112	
Monopolar prosthesis	33.3% (7)				1.17	0.44/3.10	0.10 ^NS^	0.757	
Screws	28.6% (4)				0.93	0.28/3.14	0.01 ^NS^	0.911	
Nails	30.0% (42)				1	--	--	--	--
**EMERGENCY SURGERY**									
Yes	34.4% (88)	2.83 †	0.092	0.008	2.18	0.86/5.52	2.72 ^†^	0.099	0.015
No	19.4% (6)								
**AMERICAN SOCIETY OF ANESTHESIOLOGISTS(ASA)**									
3–4	36.3% (77)	4.69 *	0.030	0.016	1.95	1.06/3.58	4.60 *	0.032	0.024
1–2	22.7% (17)								
**ANESTHESIA**									
Spinal	33.8% (78)	0.16 ^NS^	0.693	0.001	1.17	0.53/2.59	0.16 ^NS^	0.693	0.001
General	30.3% (10)								

NS = NOT significant at 10% (*p* > 0.100); ^†^ = Significant cases (*p* < 0.100); ** = Highly significant at 1% (*p* < 0.01) Spinal anesthesia: affects a part of the body and lasts between 1 and 3 h; General anesthesia: affects the whole body and lasts 24 h.

**Table 3:** Test of independence and univariate logistic regression. Effect of complication factors on the presence of delirium in octogenarian women with a hip fracture.

Factor		Description of Patients with Delirium	Contrast Test		R^2^	Univariate Logistic Regression				
			Valor	*p*		OR	95% CI	Wald	*p*	R^2^
**ANEMIA**										
	Yes	30.5% (76)	4.25 *	0.039	0.015	0.48	0.24/0.98	4.13 *	0.042	0.020
	No	47.4% (18)				1	--	--	--	--
**TRANSFUSION**										
	Yes	29.4% (32)	0.94 ^NS^	0.338	0.003	0.78	0.46/1.30	0.92 ^NS^	0.338	0.004
	No	34.8% (62)				1	--	--	--	--
**CONSTIPATION**										
	Yes	37.3% (28)	0.97 ^NS^	0.325	0.003	1.32	0.76/2.29	0.96 ^NS^	0.326	0.005
	No	31.1% (66)				1	--	--	--	--
**RESPIRATION**										
	Yes	40.4% (19)	1.50 ^NS^	0.220	0.005	1.49	0.78/2.84	1.49 ^NS^	0.222	0.007
	No	31.3% (75)				1	--	--	--	--
**ALT. RENAL FUNCTION**										
	Yes	40.9% (18)	1.57 ^NS^	0.210	0.005	1.52	0.79/2.94	1.56 ^NS^	0.212	0.007
	No	31.3% (78)				1	--	--	--	--
**URINARY TRACT INFECTION (UTI)**										
	Yes	63.4% (26)	20.42 **	0.000	0.071	4.54	2.27/9.08	18.23 **	0.000	0.090
	No	27.6% (68)				1	--	--	--	--
**MALNUTRITION**										
	Yes	32.4% (12)	0.00 ^NS^	0.965	0.000	0.98	0.47/2.06	0.02 ^NS^	0.965	0.000
	No	32.8% (82)				1	--	--	--	--
**RENAL ARTERIAL OCCLUSION (RAO)**										
	Yes	58.1% (18)	10.11 **	0.001	0.035	3.28	1.53/7.03	9.33 **	0.002	0.045
	No	29.7% (76)				1	--	--	--	--
**CHRONIC HEART FAILURE (CHF)**										
	Yes	44.8% (13)	2.14 ^NS^	0.144	0.007	1.78	0.82/3.86	2.09 ^NS^	0.148	0.010
	No	31.4% (81)				1	--	--	--	--
**ISCHEMIC CARDIOPATHY**										
	Yes	25.0% (7)	0.85 ^NS^	.357	0.003	0.66	0.27/1.61	0.84 ^NS^	0.360	0.004
	No	33.6% (87)				1	--	--	--	--

NS = NOT significant at 10% (*p* > 0.100); † = Significant cases (*p* < 0.100); ** = Highly significant at 1% (*p* < 0.01).

**Table 4:** Test of independence and multivariate logistic regression.

	B	Wald	p	R^2^ Partial	R^2^ Accumulated	OR	95% CI the OR
**Moderate cognitive impairment**	1.35	17.47 **	0.000	0.098	0.098	3.85	2.05/7.25
**Urinary tract infection**	1.34	12.05 **	0.001	0.066	0.164	3.82	1.79/8.16
**Changes in vision**	0.74	5.03 *	0.025	0.019	0.185	2.09	1.10/4.00
**Emergency intervention**	1.18	4.62 *	0.032	0.024	0.209	3.25	1.11/9.51
**Number of drugs**	−0.18	9.29 **	0.002	0.022	0.237	0.84	0.75/0.94

NS = NOT significant at 10% (*p* > 0.100); ** = Highly significant at 1% (*p* < 0.01).

**Table 5:** Test of independence and univariate logistic regression. Effect of the type of drug administered as a factor associated with an increased risk of octogenarian women with a hip fracture developing delirium.

Factor	Description of Patients with Delirium	Contrast Test		R^2^	Univariate Logistic Regression				
		Valor	*p*		OR	95% CI	Wald	*p*	R^2^
**ANTIHYPERTENSIVE**									
Yes	29.8% (62)	2.55 ^NS^	0.110	0.009	0.64	0.37/1.11	2.53 ^NS^	0.111	0.012
No	39.7% (31)				1	--	--	--	--
**BENZODIAZEPINES**									
Yes	32.5% (38)	0.00 ^NS^	0.991	0.000	1	0.60/1.65	0.00 ^NS^	0.991	0.000
No	32.5% (55)				1	--	--	--	--
**ANTIDEPRESSANT**									
Yes	33.3% (33)	0.02 ^NS^	0.879	0.000	1.04	0.62/1.75	0.02 ^NS^	0.879	0.000
No	32.4% (61)				1	--	--	--	--
**PROTON PUMP INHIBITORS**									
Yes	37.9% (33)	1.45 ^NS^	0.228	0.005	1.38	0.82/2.34	1.45 ^NS^	0.229	0.007
No	30.7% (61)				1	--	--	--	--
**ANTIAGREGANTS**									
Yes	29.2% (21)	0.56 ^NS^	0.454	0.002	0.80	0.45/1.43	0.56 ^NS^	0.454	0.003
No	34.0% (73)				1	--	--	--	--
**ANTICOAGULANTS**									
Yes	34.4% (22)	0.10 ^NS^	0.754	0.000	1.10	0.61/1.98	0.10 ^NS^	0.754	0.000
No	32.3% (72)				1	--	--	--	--
**ORAL ANTIDIABETICS**									
Yes	28.3% (15)	0.58 ^NS^	0.445	0.002	0.77	0.40/1.49	0.58 ^NS^	0.445	0.003
No	33.8% (79)				1	--	--	--	--
**ANALGESIA**									
Yes	30.6% (159	0.12 ^NS^	0.726	0.000	0.89	0.46/1.73	0.12 ^NS^	0.726	0.02
No	33.2% (79)				1	--	--	--	--

NS = NOT significant at 10% (*p* > 0.100).
